# Reproductive phase-dependent variation, sexually dimorphic expression and sex steroids-mediated transcriptional regulation of *lep* and *lepr* in lymphoid organs of *Channa punctata*

**DOI:** 10.1038/s41598-020-57922-x

**Published:** 2020-01-22

**Authors:** Amrita Bakshi, Umesh Rai

**Affiliations:** Department of Zoology, University of Delhi, Delhi, 110007 India

**Keywords:** Transcriptional regulatory elements, Ichthyology

## Abstract

The reproductive phase-dependent and sex-related differential expression of leptin (*lep*) and its receptor (*lepr*) in primary and secondary lymphoid organs of a highly nutritive economically important *Channa punctata* preempts the involvement of sex steroids in modulating intra-immuno-leptin system. This hypothesis was strengthened when plasma testosterone (T) and estradiol (E_2_) levels in male and female fish of reproductively active spawning and quiescent phases were correlated with *lep* and *lepr* expression in their immune organs. Splenic *lep* and *lepr* showed a negative correlation with T in both male and female, while with E_2_ there was a positive correlation in male and negative in female *C*. *punctata*. In head kidney, a contrasting correlation was observed as compared to spleen. To validate the implication of sex steroids in regulating leptin system in immune organs, *in vivo* and *in vitro* experiments were performed with DHT and E_2_. Upon administration, *lep* and *lepr* expression in tissues of either sex was downregulated. In addition, *in vitro* results with either of the sex steroids exemplified their direct involvement. Overall, this study, for the first time, reports correlation between sex steroids and transcript expression of leptin system in immune organs of a seasonally breeding vertebrate.

## Introduction

Leptin encoded by obese gene *Lep* also known as *Ob* is a 16 kDa non-glycosylated protein belonging to class I cytokine family and is primarily produced from adipose tissue in mammals^1^. It is reported to act through long form of membrane-bound leptin receptor (Lepr)^2^. Besides acting as a central link between feeding, adiposity and energy homeostasis^3^, leptin regulates several other physiological functions in mammals including reproduction^4^ and immunity^5^. The orthologs of *Lep* and *Lepr* have been identified in several teleosts^6–10^ in which liver, and not the adipocytes, is reported to be the major leptin-expressing organ. Regarding physiological significance, hepatic *lep* is suggested to be majorly involved in orchestration of energy trade-off rather than regulating food intake in fishes^9,11–13^. In addition to liver, expression of *lep* is shown in other tissues, including immune organs^6,10,11^. Since immune defence varies with state of reproductive activity in seasonally breeding vertebrates^14,15^, an effort needs to be made to examine correlation between leptin, immunity and reproduction.

In addition, sexual dimorphism is unveiled in levels of leptin in blood plasma^16,17^ and adipose tissue^18,19^ in mammals, being significantly higher in females than males. Interestingly, these sex-related differences in leptin protein and mRNA levels exist regardless of the amount of body fat^16^. This led to hypothesize the involvement of sex steroids in modulating leptin expression^20^. With regard to sexually dimorphic expression of leptin receptor, reports in mammals are limited and present contradictory results^21,22^. In teleosts, studies on sex-related variation in plasma levels of leptin^23,24^ or expression of *lep* and *lepr* have been meagrely explored^25,26^. In recent years, efforts have been made to understand the role of sex steroids in dimorphic variation in expression of *lep* and *lepr* in liver of fishes^27–29^. Regarding reproductive phase-dependent expression of *lep* and its receptor, a single study is available in teleost^30^. However, sex-related reproductive state-dependent expression of leptin and leptin receptor and their transcriptional regulation remain unexplored in immune organs of vertebrates in spite of the fact that leptin is reported to coordinate seasonal immune responses as a neuroendocrine mediator^31^. In view of this, the present study was aimed to investigate the differential expression of *lep* and *lepr* in primary as well as secondary lymphoid organs, head kidney and spleen, respectively, depending on reproductive phases in both the sexes of spotted snakehead *Channa punctata*. Also, attempt was made to demonstrate sex-related variation in expression of *lep* and *lepr*, and their transcriptional regulation by sex steroids in the immune organs of *C. punctata*.

## Results

### Reproductive phase-dependent expression in male and female lymphoid organs

#### Spleen

**Figure 1:**
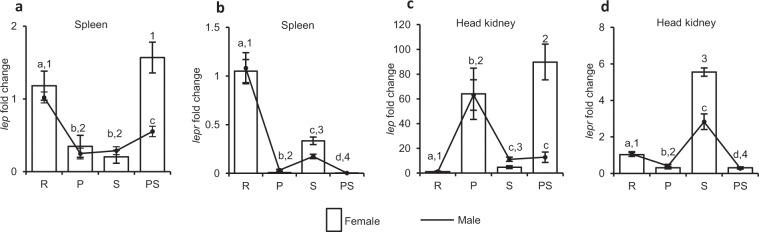
Expression of *lep* and *lepr* during different reproductive phases (R: resting, P: preparatory, S: spawning and PS: postspawning) in lymphoid organs of male and female *C. punctata*. (**a**,**b**) Showing expression of *lep* and *lepr*, respectively in spleen while (**c**,**d**) is denoting their expression in the head kidney. Relative fold change was calculated considering resting phase as reference. One-way analysis of variance (ANOVA, p < 0.0001, N = 8 for each tissue of either sex in each reproductive phase) followed by Student’s Newman Keuls (SNK) post-hoc test (p < 0.05) was used to determine the significant difference between the groups. Different superscripts (alphabets for male and numerals for female) above error bars indicate significant differences between the groups. The data is represented as mean fold change ± standard error of mean (SEM).

The expression of *lep* and *lepr* during different reproductive phases in both, male and female *C. punctata*, exhibited marked variation depending on their reproductive state (one-way analysis of variance, ANOVA, p* < *0.0001; Fig. 1a,b). In male, lowest *lep* transcripts level was observed during preparatory and spawning phases. Compared to these reproductively active phases, *lep* expression was considerably (p < 0.05) higher in reproductively quiescent phases, *i.e*., postspawning phase and resting phase. An appreciable (p* < *0.05) increase in its expression observed during postspawning phase further increased significantly (p* < *0.05) in resting phase (Fig. 1a). The *lep* expression in female was largely comparable to male, considerably (p* < *0.05) lower during reproductively active than quiescent phases (Fig. 1a). In case of phase-dependent expression of *lepr*, unlike *lep*, minimal expression was recorded during postspawning phase in both the sexes (Fig. 1b). Nonetheless, maximum *lepr* transcript level was observed during resting phase in male as well as female. After resting phase, a sharp (p* < *0.05) decline in *lepr* expression was detected during preparatory phase. In spawning phase, an appreciable (p* < *0.05) increase in *lepr* level was observed compared to preparatory phase, though the level was significantly (p* < *0.05) lower than resting phase in both the sexes.

#### Head kidney

Although expression of *lep* and *lepr* in head kidney of male and female *C*. *punctata* showed significant variation depending on reproductive phases (one-way ANOVA, p* < *0.0001; Fig. 1c,d), their expression pattern was different with that of spleen. The lowest expression of *lep* was observed during resting phase in both male and female *C*. *punctata* (Fig. 1c). Thereafter, *lep* expression highly (p* < *0.05) increased during preparatory phase in both the sexes. Although level of *lep* declined considerably (p* < *0.05) during spawning phase in male as well as female, it remained unaltered until postspawning phase in male while significantly (p < 0.05) increased in female. Regarding reproductive phase-dependent expression of *lepr* (Fig. 1d), the expression pattern in both the sexes was found to be quite similar, with maximum transcript levels during spawning phase and minimum during preparatory and postspawning phases. In resting phase, *lepr* expression was considerably (p* < *0.05) higher than preparatory and postspawning phases but significantly (p* < *0.05) lower than spawning phase.

### Sexual dimorphism in expression of *lep* and *lepr* in lymphoid organs

**Figure 2:**
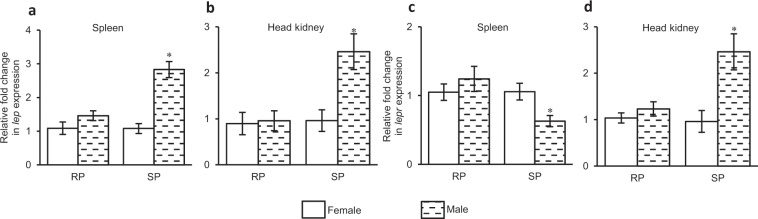
Sex-related differential expression of (**a**,**b**) *lep* and (**c**,**d**) *lepr* in lymphoid organs during reproductively quiescent resting phase (RP) and active spawning phase (SP) of *C. punctata*. The relative fold change was calculated considering female as reference. Student’s unpaired t-test was employed and graphs are represented as mean fold change ± SEM (N = 8). Asterisk ‘*’ above error bars indicates significant (p < 0.02) difference in gene expression in a lymphoid tissue of male and female during same reproductive phase.

Sex-related differential expression of *lep* and *lepr* in spleen and head kidney during spawning and resting phases showed marked (p* < *0.02) difference in their transcripts level in both the lymphoid organs only during reproductively active spawning phase (Fig. 2). During active phase, expression of *lep* was 2.5 to 3-fold higher in spleen (p < 0.0001) and head kidney (p = 0.0052) of male than that in respective tissues of female *C. punctata* (male *vs* female lymphoid tissue, unpaired t-test, Fig. 2a,b). Regarding sexually dimorphic expression of *lepr*, contrasting results were observed between primary and secondary lymphoid organs. In case of spleen, transcript level of *lepr* was considerably (~0.4 fold, p = 0.0115) higher in female than male while in head kidney it was 2.5-fold (p = 0.0012) higher in male than female fish (Fig. 2c,d). Unlike reproductively active phase, no sex-related difference in mRNA levels of *lep* and *lepr* was observed during reproductively quiescent resting phase in any lymphoid organ (Fig. 2).

### Plasma level of sex steroids in reproductively active and quiescent phase female

The level of sex steroids, 17β-estradiol (E_2_) and testosterone (T), in female *C. punctata* was considerably (unpaired t-test, p < 0.01, Table 1) higher in reproductively active spawning phase as compared to quiescent resting phase.

**Table 1:** Plasma levels of 17β-estradiol (E_2_) and testosterone (T) in adult female *C. punctata*.

Reproductive phase	E_2_ level (mean ± SEM in pg/ml)	T level (mean ± SEM in pg/ml)
Spawning (reproductively active)	987.0269 ± 179.70	207.6 ± 53.38
Resting (reproductively quiescent)	134.1134 ± 5.910	31.61 ± 0.5421

### Correlation analyses

#### Correlation of lep and lepr expression with sex steroids

**Figure 3:**
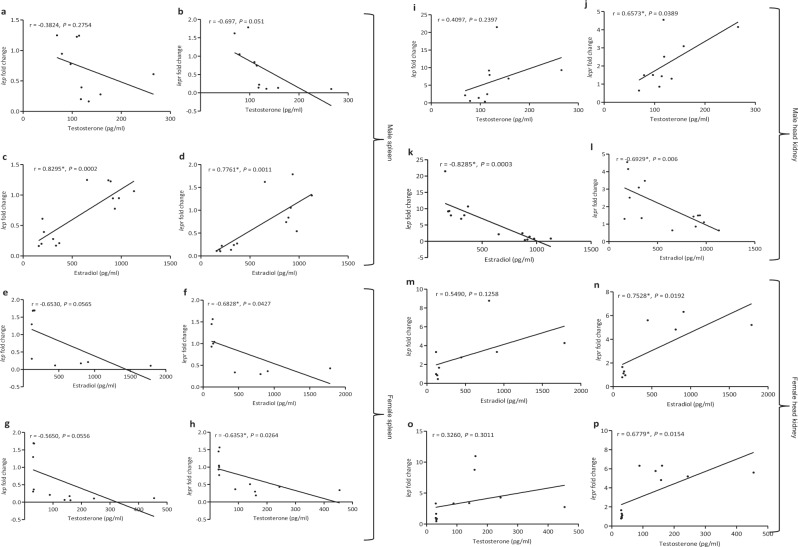
Showing correlation of *lep* and *lepr* expression in lymphoid organs (**a**–**h**) spleen; (**i**–**p**) head kidney of male and female with levels of their plasma sex steroids, testosterone (T) and 17β-estradiol (E_2_), in resting and spawning phases. The significant (p < 0.05) Pearson’s correlation coefficient (r) value is marked by asterisk (*).

The plasma levels of sex steroids, T and E_2_, in male and female *C. punctata* during reproductively active spawning and quiescent resting phases correlated with expression of *lep* and *lepr* in spleen and head kidney showed differential results depending on sex, specific sex steroid and type of lymphoid tissue (Fig. 3). In male, a strong positive correlation was observed between level of plasma E_2_ and expression of splenic *lep* (r = 0.8295, p = 0.0002) as well as *lepr* (r = 0.7761, p = 0.0011) (Fig. 3c,d) while an insignificant negative correlation was seen between plasma T and splenic leptin system (*lep*: r = −0.3824, p* = *0.2754; *lepr*: r = −0.697, p* = *0.051; Fig. 3a,b). Contrary to spleen, a marked negative correlation between plasma E_2_ levels and *lep* (r = −0.8285, p = 0.0003) / *lepr* (r = −0.6929, p = 0.006) expression (Fig. 3k,l) while a positive correlation between T and *lep* (r = 0.4097, p = 0.2397) / *lepr* (r = 0.6573, p = 0.0389) was observed in head kidney (Fig. 3i,j). In case of female, plasma levels of both the sex steroids exhibited a negative correlation with splenic *lep* and *lepr* (E_2_-*lep*: −0.6530, p = 0.0565; E_2_-*lepr*: r = −0.6828, p = 0.0427; T-*lep*: r = −0.5650, p = 0.0556; T-*lepr*: r = −0.6353, p = 0.0264; Fig. 3e–h) while a positive correlation with expression of leptin system in head kidney (E_2_-*lep*: r = 0.5490, p = 0.1258; E_2_-*lepr*: r = 0.7528, p = 0.0192; T-*lep*: r = 0.3260, p = 0.3011; T-*lepr*: r = 0.6779, p = 0.0154; Fig. 3m–p).

#### Correlation between expression of *lep* and its receptor (*lepr*)

No significant correlation was observed between relative expression of *lep* and *lepr* in any of the immune organs of either male or female *C. punctata* during different reproductive phases (Supplementary Table S3).

### Role of sex steroids in regulation of *lep* and *lepr* expression in lymphoid organs

#### *In vivo* experiment

**Figure 4:**
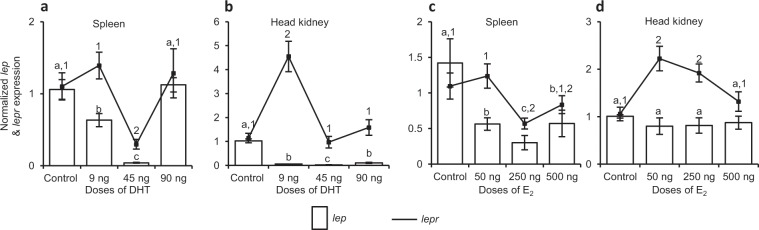
*In vivo* effect of different doses of (**a**,**b**) dihydrotestosterone (DHT) and (**c**,**d**) E_2_ on *lep* and *lepr* expression in lymphoid organs of male and female *C. punctata*, respectively (N = 8 for each dose of sex steroid in male or female). The control groups received comparable volume of vehicle. The expression was calculated in fold change and expressed as mean ± SEM. Data in fold change expression was analysed by one-way ANOVA and compared by SNK test. Different alphabets or numerals on error bars indicate significant (p < 0.05) difference between the expression of *lep* or *lepr*, respectively, in different groups.

Effect of dihydrotestosterone (DHT) on *lep* and *lepr* expression in male fish. The male *C. punctata* receiving varying doses of DHT (9, 45 and 90 ng per day/fish for 3 days) during resting phase exhibited dose- and tissue-related differential effect of non-aromatizable androgen on *lep* and *lepr* expression in spleen and head kidney (ANOVA, p* < *0.01, Fig. 4a,b). Compared to splenic *lep* expression in vehicle-injected control, a marked (p* < *0.05) decrease in its expression was observed after treatment with low dose (9 ng/fish/day) of DHT. The DHT-induced down-regulation became severely pronounced with an increase of its dose to 45 ng/fish/day (9 ng *vs* 45 ng, p* < *0.05; Fig. 4a). However, the highest dose of DHT (90 ng/fish/day) was seen ineffective in influencing splenic *lep* expression. In case of head kidney, all the doses of DHT were effective in significantly (p* < *0.05) reducing *lep* expression, though maximal inhibition was observed at the moderate dose of 45 ng/fish/day (Fig. 4b). Unlike *lep* that was inhibited by DHT in both the lymphoid organs, expression of *lepr* after DHT treatment showed contradictory results, a marked (p* < *0.05) decline in spleen while a robust (p* < *0.05) increase in head kidney. However, only the moderate dose of DHT (45 ng/fish/day) was found to be effective in modulating *lepr* expression in spleen whereas the lowest dose (9 ng/fish/day) in case of head kidney (Fig. 4).

Effect of 17β-estradiol (E_2_) on *lep* and *lepr* expression in female fish. Treatment of female *C. punctata* with different doses of E_2_ during resting phase resulted in a marked (one-way ANOVA, p = 0.0035) decrease of *lep* expression in spleen at all the doses (50, 250 or 500 ng E_2_/fish/day for 3 days), though a significant (p < 0.05) decline in splenic *lepr* was observed only at the dose of 250 ng/fish/day when compared to that of vehicle-injected female control (Fig. 4c). Unlike spleen, in head kidney E_2_ treatment failed to affect *lep* expression (p < 0.05) at any of its dose while considerably (one-way ANOVA, p = 0.0012) increased *lepr* expression at the doses of 50 and 250 ng/fish/day (Fig. 4d).

**Figure 5:**
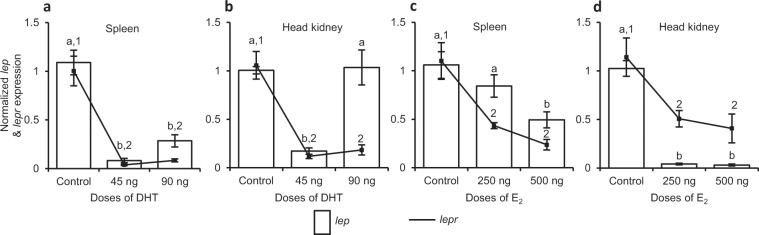
Criss-cross *in vivo* experiments showing effect of (**a**,**b**) male sex steroid DHT in female and (**c**,**d**) female sex steroid E_2_ in male, on *lep* and *lepr* expression in lymphoid organs (N = 8 for each dose of sex steroid in male or female). Values expressed in fold change (mean ± SEM) bearing different superscripts (alphabets for *lep* and numerals for *lepr*) on error bars differ significantly (one-way ANOVA followed by SNK test, p < 0.05).

Criss-cross experiment: effect of DHT in female and E_2_ in male fish. The administration of DHT in female fish of resting phase led to a significant (p < 0.0001) decrease of *lep* as well as *lepr* expression in both the immune organs, spleen and head kidney when compared to vehicle-injected female control, with an exception at the dose of 90 ng/fish/day for *lepr* in head kidney where no marked alteration in its expression was observed (Fig. 5a,b). Like inhibitory effect of the DHT in female, E_2_ administration in male caused a marked (p* < *0.01) decline in *lep* and *lepr* expression in spleen as well as head kidney when compared to their expression in respective immune organ of vehicle-injected male fish of resting phase (Fig. 5c,d).

#### *In vitro* experiment

**Figure 6:**
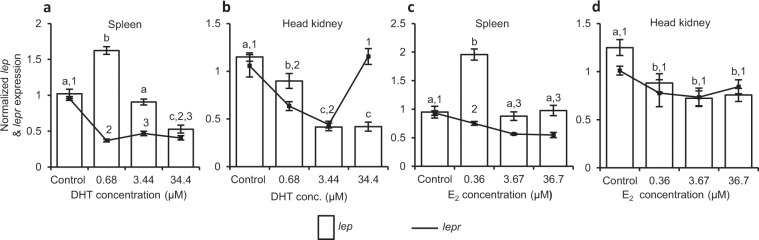
*In vitro* effect of different concentrations of (**a**,**b**) DHT and (**c**,**d**) E_2_ on *lep* and *lepr* expression in lymphoid organs of male and female *C. punctata*, respectively. The culture was run in hexaplicate (N = 6) for 12 h. The expression is shown as mean fold change ± SEM where different superscripts (alphabets for *lep* and numerals for *lepr*) on error bars indicate significant (p < 0.05) difference between the groups (one-way ANOVA followed by SNK test).

Effect of DHT on male lymphoid organs. The pieces of spleen incubated with different concentrations of DHT (p* < *0.0001) showed concentration-related dual effects on *lep* expression, stimulatory (p < 0.05) at the lowest concentration while inhibitory (p < 0.05) at the highest concentration when compared to that incubated in medium alone (control). However, on splenic *lepr* expression, DHT had marked (p < 0.0001) inhibitory effect at all the concentrations (Fig. 6a). In case of head kidney, expression of both, *lep* and *lepr*, considerably (p < 0.0001) decreased after incubation with varying concentrations of DHT, except 34.4 µM for *lepr* (Fig. 6b).

Effect of E_2_ on female lymphoid organs. The expression of *lep* and *lepr* in spleen and head kidney incubated with varying concentration (0.36, 3.67 and 36.7 µM) of E_2_ exhibited differential results (Fig. 6c,d). In spleen, E_2_ significantly (p < 0.05) inhibited the expression of *lepr* at all the concentrations while it appreciably (p < 0.05) augmented the expression of *lep* at the lowest concentration and failed to alter at the subsequent higher concentrations (Fig. 6c). In contrast to spleen, in head kidney E_2_ markedly (p < 0.05) reduced *lep* expression at all the concentrations though failed (p = 0.2039) to affect *lepr* expression at any of its concentration (Fig. 6d).

**Figure 7:**
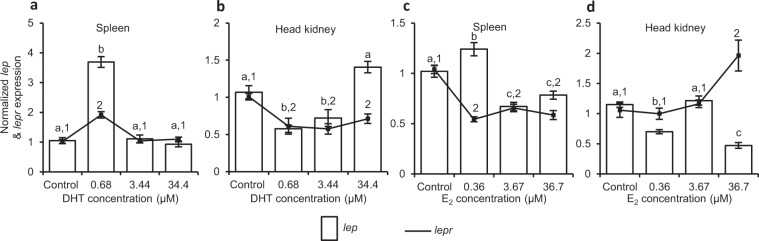
Criss-cross *in vitro* experiments where (**a**,**b**) splenic and head kidney tissues from female were incubated with different concentrations of DHT while (**c**,**d**) male tissues were incubated with varying concentrations of E_2_. For each concentration of DHT/E_2_/control, culture was run in hexaplicate (N = 6). Effect of sex steroids on *lep* and *lepr* expression in spleen and head kidney of both the sexes are expressed in fold change. Data were analysed by one-way ANOVA, compared by SNK test and represented as mean ± SEM. Values with different superscripts (alphabets for *lep* and numerals for *lepr*) indicate significant (p < 0.05) differences between the groups.

Criss-cross experiment: effect of DHT on female and E_2_ on male lymphoid organs: The fragments of lymphoid organs from female incubated with different concentrations (0.68, 3.44 and 34.4 µM) of DHT showed an appreciable (p < 0.05) increase in splenic *lep* and *lepr* expression only at the lowest concentration (Fig. 7a) as their expression at subsequent higher concentrations were comparable to that incubated in medium alone (control). Unlike spleen, DHT significantly inhibited the expression of both, *lep* (p < 0.0001) and *lepr* (p = 0.0013), in head kidney (Fig. 7b). In case of male, pieces of lymphoid organs incubated with varying concentrations of E_2_ exhibited dual effects (p < 0.0001) on splenic *lep* expression, stimulatory at lower concentration (0.36 µM; p < 0.05) while inhibitory at higher concentrations (3.67 and 36.7 µM; p < 0.05). However, expression of splenic *lepr* decreased considerably (p < 0.0001) at all the concentrations of E_2_ when compared to control (Fig. 7c). With regard to expression of leptin system in head kidney, an inconsistent effect of E_2_ was observed on *lep* expression, inhibitory (p < 0.05) at 0.36 and 36.7 µM concentrations while no effect at 3.67 µM. On *lepr*, an appreciable (p < 0.05) increase was recorded at the highest concentration of E_2_ (Fig. 7d).

## Discussion

The present study aimed to investigate the reproductive phase-dependent and sex-related variations in expression of leptin and leptin receptor in primary and secondary lymphoid organs, head kidney and spleen, respectively, of an adult *Channa punctata*. The sexually dimorphic expression of *lep* and *lepr* during reproductively active but not during the quiescent phase, led the authors to hypothesize the involvement of sex steroids in modulating transcripts level of leptin and its receptor. Therefore, *in vivo* and *in vitro* experiments were undertaken to elucidate the role of dihydrotestosterone and 17β-estradiol on the expression of *lep* and *lepr* in spleen as well as head kidney of adult male and female *C. punctata*.

The expression of leptin and its receptor has been demonstrated in lymphoid organs of a few fishes^10,11,32,33^. Likewise, an intense expression of *lep* and *lepr* was observed in spleen and head kidney of *C. punctata* though the pattern varied from secondary to primary lymphoid organ. Recent studies in fishes have also shown marked difference between spleen and head kidney with respect to expression pattern of immune-related genes and their respective proteins in response to bacterial infection^34–36^. These differences have been attributed to differential role of primary and secondary lymphoid organs as teleostean spleen is proposed to be majorly involved in cellular responses while head kidney in humoral responses^34^. Another possibility could be due to differences in mRNA turnover of *lep* and *lepr* between two lymphoid organs as mRNA turnover has been suggested to be dependent on RNA binding proteins (RBPs) and expression of these RBPs is reported to vary from tissue to tissue in humans^37^. Nonetheless, expression of *lep* and *lepr* in spleen and head kidney point towards the localized role of leptin system in fish immune organs. Till date, only two studies have been conducted in fishes highlighting the direct implication of leptin in immune responses. Leptin knockdown in zebrafish caused an increase in bacterial load due to marked reduction in ability to fight pathogens^38^. Another study reports decreased superoxide production from blood leucocytes of trout when incubated with homologous leptin under *in vitro* condition^39^. Moreover, in mammals, role of leptin in regulating immune functions is well demonstrated where it is shown to modulate both the arms of immunity, innate as well as adaptive^5^. Taken together, presumptive immunomodulatory role of leptin is speculated in *C. punctata*.

Interestingly, *lep* and *lepr* expression during different reproductive phases has not been estimated so far in lymphoid organs of fishes or any other seasonally breeding vertebrate despite the fact that immune defence process varies depending on season^14,15^. The current study for the first time reports reproductive phase-dependent variation in *lep* and *lepr* expression in lymphoid organs of vertebrates. In spleen of male *C. punctata*, expression of *lep* remained considerably low during preparatory and spawning phases while high in resting and postspawning phases. The reproductive phase-dependent pattern of splenic *lep* expression in female *C. punctata* was largely comparable to that of male. Also, male and female *C. punctata* exhibited similar expression pattern for splenic *lepr*, being high in resting and low in preparatory and postspawning phases. In case of male head kidney, level of *lep* remained low during resting, spawning and postspawning phases while substantially high during preparatory phase. The pattern of *lep* expression in head kidney of female during different reproductive phases was similar to that of male, except postspawning phase. Regarding head kidney *lepr*, the expression pattern in both the sexes was found to be identical along the reproductive phases. These observations in the present study led to speculate a correlation between reproductive phase-dependent variations in expression of *lep* and *lepr* in lymphoid organs and relative concentration of androgen and estrogen in male and female *C*. *punctata*. In male *C. punctata*, a peak of T and 11-ketotestosterone was attained during preparatory phase while E_2_ in resting phase^40^. Both T and E_2_ remained high during preparatory phase of female *C. punctata*. Further, our assumption gets support from studies in mammals where male and female sex steroids are shown to have opposite effects on leptin production from adipose tissue^17,18,41^. As far as interrelation between ligand and its receptor expression is concerned, *lep* did not correspond to *lepr* in both male and female *C*. *punctata* depending on reproductive phase and lymphoid tissue. Similar to the findings of present study, expression pattern of *lep* has been reported to be opposite to *lepr* in liver of *Oryzias latipes*^8^, *Salmo salar*^42^, *Pelteobagrus fulvidraco*^11^, *Epinephelus coioides*^33^ and *Oreochromis niloticus*^43^. Although possible explanation for opposite expression pattern of *lep* and *lepr* has not been proposed in these studies, one could speculate the implication of an unknown receptor-mediated mechanism as suggested by Farooqi and colleagues^44^. They observed that leptin-deficient patients show more severe phenotypes as compared to leptin receptor-deficient ones. Moreover, difference between half-life of ligand and its receptor mRNA and/or alteration in binding affinity of leptin receptor depending on reproductive phase could be taken into account for reverse pattern between ligand and its receptor during postspawning phase in *C. punctata*.

The sexual dimorphism in plasma levels of leptin^17–19,45,46^ and its soluble receptor^46^ is well demonstrated in mammals. Regarding their sex-related expression in tissues, studies are largely confined to adipocytes^19,45^, skeletal muscle^46^ and hypothalamus^22^. The levels of leptin and its receptor in these studies are reported to be higher in females than males. However, no attempt has been made to investigate the sexually dimorphic expression of *LEP* and *LEPR* in immune organs of mammals. In fishes, a handful of studies are available on sex-related dimorphism in levels of leptin and its receptor, and the results are inconsistent^23–26^. The plasma levels of leptin in *Lota lota* is shown to be high in female as compared to male before, during and after spawning season^23^ while no sex-related difference in plasma leptin levels is reported in *Cyprinus carpio* and *Capoeta trutta*^24^. It is to be noted that the reproductive phase during which plasma leptin was assayed in *C*. *carpio* and *C*. *trutta* is not highlighted in the report. The efforts have also been made to examine sex-related differential expression of *lep* and *lepr* in various tissues (brain, liver, hypothalamus, pituitary and gonads) during different stages of germ cell development in male and female *Megalobrama amblycephala*^25^. The results varied depending on tissues and stages of spermatogenesis/folliculogenesis. Similarly, tissue-wise sex-related marked difference in *lep* expression is demonstrated in several tissues (brain, liver, gills, intestine, kidney, heart, muscle and spleen) but adipocytes in sexually immature, and also in gonads of sexually mature *Tanichthys albonubes*^26^. This is the only study in fishes that reports sexual dimorphism in expression of *lep* in a lymphoid organ in which leptin transcript level is shown to be high in male than female^26^. However, sex-related differential expression of *lepr* has not been explored so far in primary or secondary lymphoid organs of fishes. In the current study, sexually dimorphic expression of *lep* and *lepr* was observed in spleen as well as head kidney of *C. punctata* during reproductively active phase when level of sex steroids remain high and not in quiescent phase when their levels remain basal. As observed in immature *T. albonubes*^26^, splenic *lep* was high in male than female *C. punctata*. A similar pattern of *lep* expression was also seen in head kidney. However, a contrasting expression pattern of its receptor was observed between primary and secondary lymphoid organs of *C. punctata* as level of *lepr* was recorded higher in spleen of female while head kidney of male. Taken together, we speculated the involvement of sex steroids in sexually dimorphic expression of *lep* and *lepr* in immune organs of *C. punctata*.

In the present study, role of sex steroids in control of leptin and its receptor expression in immune organs of *C. punctata* was deduced based on observations during different reproductive phases in both the sexes and also between opposite sexes in the same reproductive phase. This inference was backed by correlation analysis between levels of plasma sex steroids and *lep* as well as *lepr* expression in immune organs during reproductively active and quiescent phases in male and female *C*. *punctata*. Although efforts have not been made to examine an interrelationship between levels of plasma sex steroids and leptin system in immune organs of seasonally breeding vertebrates, studies in mammals have suggested that plasma leptin is negatively associated with androgens in males^47,48^ while positively with estradiol in females^49^. In male *C*. *punctata*, a positive correlation between levels of plasma T and *lep* and *lepr* was observed in primary lymphoid organ, *i.e*., head kidney while negative in case of secondary lymphoid organ, *i.e*., spleen. The relationship with E_2_ in male immune organs was contradictory to T, negative with *lep* and *lepr* in head kidney while positive with that in spleen. Interestingly, in female *C*. *punctata*, *lep* and *lepr* expression in spleen exhibited negative correlation with both T and E_2_ and *vice versa* in head kidney. This implies that *lep* and *lepr* expression in immune organs depends on sex, prevalence of sex steroids and type of lymphoid organ.

The inference drawn from correlation analyses gets support from our *in vivo* study, including criss-cross experiments in which male and female *C. punctata* received E_2_ and DHT, respectively. Sex steroids, depending on dose, had either inhibitory or no effect on *lep* and *lepr* expression in primary as well as secondary lymphoid organs of both the sexes, except on *lepr* in head kidney where DHT had marked stimulatory effect in male and E_2_ in female. Since the effect of sex steroids on *lep* and *lepr* expression remain unexplored in immune organs of vertebrates, we analysed our results in light of observations in other tissues of fishes^26,28,29^ and mammals^41,50–54^. Our findings on *lep* and *lepr* expression in immune organs of *C. punctata* are largely contrasting to a recent study in immature male *Salmo salar* in which testosterone is reported to increase *lep* expression in liver and pituitary while plasma level of leptin has been shown to remain unchanged in androgen-treated fish^28^. In another study, estradiol has been shown to upregulate hepatic *lep* expression in a dose- and time-dependent manner in immature female as well as male *T. albonubes*^26^. Regarding leptin receptor, testosterone is reported to stimulate the transcription in pituitary but not in testis of immature male *S. salar*. However, 11-ketoandrostenedione had no effect on the expression of both, *lep* and its receptor, in any of the tissues^28^. In mammals, male and female sex steroids have contrasting effects on expression and production of leptin from adipose tissue, inhibitory effect of androgen in male as well as female^17,18^ while stimulatory effect of estrogen in female^41^. With regard to sex steroid-induced modulation of *LEPR* expression, studies are meagre and restricted to estrogen only. Estradiol is reported to stimulate hypothalamic *LEPR* expression in rat^50^ but not in heifers^51^. Also, effect of E_2_ on the receptor expression is shown to vary with tissues in heifers as it has been inhibitory in uterine endometrium and mammary adipose tissue while ineffective in liver, muscle and subcutaneous adipose tissue^51^. Based on these studies and our observations in *C. punctata*, it is evident that role of sex steroids in modulation of leptin and its receptor expression vary with sex, tissue and species.

In addition, *in vitro* experiments with DHT and E_2_ in the present study exemplifies direct implication of sex steroids in regulation of leptin and its receptor expression in lymphoid organs of male and female *C. punctata*. Although no such *in vitro* experiments are conducted with immune tissues of fishes or other vertebrates, culture of hepatocytes with androgen and estrogen, separately, has shown upregulation of *lep* expression in immature/adult male and immature female *S. salar*^27^. In contrast, male as well as female sex steroids have been demonstrated to downregulate the expression of *lep* and its receptor in hepatocytes of immature female *Cynoglossus semilaevis*^31^. Apart from fishes, direct role of sex steroids has been explored also in mammals where androgens and estrogen are shown to have differential effects, inhibitory, stimulatory or no effect, on leptin production from adipocytes^18,52–54^. Surprisingly, *in vitro* study to demonstrate the effect of sex steroids on expression of leptin receptor is missing in mammals. In the current *in vitro* study, DHT and E_2_ downregulated the transcription of leptin and its receptor in spleen and head kidney of *C. punctata*, except DHT on *lep* and *lepr* in female spleen and E_2_ on *lep* in female spleen and *lepr* in male head kidney where upregulation in transcription was observed. Also, dual effects of sex steroids depending on concentration were observed on *lep* expression in male spleen. This largely ratify the *in vivo* observations that have evidenced variable role of sex steroids depending on dose/concentration, type of lymphoid organs and sex of fish, in modulating the leptin system.

## Conclusion

The expression of *lep* and *lepr* in immune organs of *C. punctata* preempts the direct role of leptin in regulation of immune system in fishes. Also, seasonal variation in expression of *lep* and *lepr* in primary as well as secondary lymphoid organs of *C*. *punctata* point towards an axis operating between reproduction-leptin-immunity in fishes. A marked sexual dimorphism in splenic and head kidney *lep* and *lepr* expression during reproductively active phase only suggested the involvement of sex steroids in regulating leptin system of lymphoid organs. This hypothesis was validated by *in vivo* and *in vitro* experiments with sex steroids. In view of this, sex steroids emerge as important modulator of leptin system in lymphoid organs of fishes.

## Materials and Methods

### Reagents and culture medium

The culture medium RPMI 1640 was supplemented with 0.1 mg/ml streptomycin sulphate and 40 µg/ml gentamycin sulphate. Stock solution of sex steroids, 5α-dihydrotestosterone (DHT) and 17β-estradiol 3-benzoate (E_2_), were made in ethanol (1 mg/ml). Their further dilutions ranging from 10 µg/ml to 100 ng/ml were prepared in phosphate buffered saline (PBS, pH 7.4) containing above said concentrations of streptomycin and gentamycin. Finally, working concentrations of sex steroids were made in culture medium. The media of control groups contained the maximum concentration of ethanol (0.001%). Estradiol and testosterone enzyme-linked immunosorbent assay (EIA) kits were purchased from Cayman Chemical.

### Animals and ethics statement

Fishes (90–120 g) were procured from wild population (National Capital Region of Delhi) and acclimated to laboratory conditions for a week at 12 L:12 D prior to experiments. They were fed *ad libitum* with minced chicken liver. An overdose of 2-phenoxyethanol (5 ml/L water) was used to sacrifice the fish. Our protocol has been approved by the Institutional Animal Ethics Committee (DU/ZOOL/IAEC-R/2017/06), Department of Zoology, University of Delhi. All experiments were carried out following relevant guidelines and regulations of IAEC.

### Differential expression of *lep* and *lepr*

#### Reproductive phase-dependent

On the basis of histological observations of gonads, reproductive cycle has been broadly delineated into resting, preparatory, spawning and postspawning phases in male^40^ and female^55^ *Channa punctata*. Fishes were procured during the peak of each reproductive phase to examine differential expression of leptin (*lep*) and leptin receptor (*lepr*) in primary and secondary lymphoid organ, head kidney and spleen, respectively. In each reproductive phase, spleen and head kidney from male and female fish (N = 8 for each sex) were dissected out, quickly frozen in liquid nitrogen and stored at −80 °C until RNA extraction.

#### Sex-dependent

To examine sex-related differential expression of *lep* and *lepr* in lymphoid organs, data of contrasting reproductive phases, spawning (active) and resting (quiescent), were selected. The expression of *lep* and *lepr* in spleen or head kidney of female during resting or spawning phase was compared with respective gene expression in the same lymphoid organ of male of that particular phase.

### Experiment: Effect of sex steroids on *lep* and *lepr* expression in lymphoid organs

To examine the role of sex steroids in regulation of *lep* and *lepr* expression in spleen and head kidney, *in vivo* and *in vitro* experiments were performed with sex steroids during resting phase when plasma T and E_2_ remain at basal level in male and female *C. punctata*, respectively.

#### *In vivo* experiment

The range of doses for DHT and E_2_ was determined based on plasma levels of T and E_2_ in adult male and female *C. punctata*, respectively, during different reproductive phases. Each male fish of group I, II and III received 9, 45 and 90 ng of DHT/injection/day, respectively. Likewise, three groups of female fish were made to receive different doses of E_2_ (50 ng, 250 ng and 500 ng per injection/day/fish).

Given the facts that female sex steroid E_2_ plays critical role in regulation of male reproduction^40,56^ and *vice versa* for testosterone in female fish^57,58^, a criss-cross experiment was designed where male fish received different doses of E_2_ (250 and 500 ng/injection/fish/day) while females were administered 45 and 90 ng of DHT/injection/fish/day. For controls, fishes of both the sexes were injected with comparable volume of vehicle (100 µl of 0.6% saline/injection/fish/day).

Fish of all the groups (N = 8 for each experimental group of male or female) received injections for three consecutive days. They were sacrificed after 18 h of the last injection. Their spleens and both side head kidneys were dissected out and used for gene expression analysis.

#### *In vitro* experiment

The experiment was designed to determine the direct role of sex steroids in regulation of *lep* and *lepr* expression in immune organs. Spleens and both side head kidneys from six adult male and same number of female *C. punctata* were dissected out, pooled sex-wise, washed and chopped into small pieces. Prior to incubation with sex steroids, tissue (10–15 mg/well) from spleen or head kidney was cultured in medium for 2 h in a 24-well culture plate. Thereafter, spent media was aspirated out and fresh media containing different concentrations of E_2_ (0.36 µM, 3.67 µM, 36.7 µM) or DHT (0.68 µM, 3.44 µM, 34.4 µM) were added to each well of culture plate. For controls, tissues were incubated in medium alone. The culture was run in hexaplicate. After12 h of incubation, tissues were collected, zap frozen in liquid nitrogen and stored at −80 °C until RNA extraction.

### Relative gene expression using quantitative real-time PCR

Total RNA was extracted from spleen and head kidney using TRI reagent following manufacturer’s instructions. RNA samples having optical density ratio (A_260/280_) between 1.8 to 2.0 and optimal integrity were processed for cDNA preparation. In brief, one microgram RNA was incubated with 10U DNase I at 37 °C for 30 min. Thereafter, DNase was heat-inactivated at 65 °C for 10 min in the presence of EDTA (50 mM, pH 8.0). Further, random hexamer primer was used to reverse transcribe the DNase-treated RNA. The cDNA thus prepared was validated by reverse transcription Polymerase Chain Reaction (RT-PCR) using a housekeeping gene 18S ribosomal RNA (*18S rRNA*). Primers specific for leptin and leptin receptor (Supplementary Table S1) were designed using testicular transcriptome data of *C*. *punctata* (NCBI bioproject accession no. PRJNA304088). The obtained sequences (accession number: *lep*- MK039679, *lepr*- MK039680) were used to design the primers for quantitative PCR (qPCR, Supplementary Table S2). The percentage efficiency of qPCR primers is also listed in Supplementary Table S2. In parallel, as a reference gene, specific primers of *18S rRNA* were used for qPCR. The reaction was carried out using power SYBR Green PCR Master Mix following manufacturer’s protocol. All the samples were run in duplicate and no template control were run with each reaction.

### Estimation of plasma sex steroids

For estimation of E_2_ and T during reproductively active spawning and quiescent resting phases in female fish (N = 6 for each reproductive phase), blood was collected and centrifuged at 2300 g for 10 min. Plasma was extracted out and stored at −80 °C until steroid estimation. To extract the total steroid, 1 ml diethyl ether was added to 200 µl plasma sample, vortexed for 5 min, kept at room temperature for 15 min and ether phase was separated out. This procedure was repeated thrice to maximally extract total steroid from the same aliquot of a plasma sample. The collected ether phases were pooled, evaporated at room temperature, dried and kept at −20 °C until assayed for E_2_ and T. Each sample was reconstituted in 200 µl of assay buffer and loaded in duplicates for estimation of E_2_ and T using respective EIA kit. As per manufacturer’s protocol, minimum detection limit of E_2_ and T was 19 and 6 pg/ml, respectively. The accuracy of kit was verified by percentage recovery and linearity of detection using serial dilutions of sample.

Our earlier report on plasma level of sex steroids in male *C. punctata*^53^, showing high level of T and low level of E_2_ during spawning than resting phase, was used in the present study for correlation analysis.

### Correlation analyses

A correlation between expression of *lep* and *lepr* in lymphoid organs and plasma level of sex steroids was examined in male as well as female *C. punctata* during reproductively active and quiescent phases, spawning and resting, respectively. To understand immune organ-specific reproductive phase-dependent interrelation between expression of ligand and its receptor, a correlation analysis was also carried out between expression of *lep* and *lepr* in each lymphoid organ of male as well as female *C. punctata* along their reproductive phases.

### Statistical analysis

After normalization with *18S rRNA* expression, relative fold change was calculated following an optimized method^59^. In case of *lep* expression in head kidney of male and female, fold change values were log-transformed to meet normality and heterogeneity of variance. One-way analysis of variance followed by Student-Newman-Keuls (SNK) multiple range test was employed to analyse expression of *lep* and *lepr* in each lymphoid organ of either sex depending on reproductive phases or after treatment (*in vivo*/*in vitro*) with sex steroids. Student’s unpaired t-test was applied for sex-related differential expression (male *vs* female, p < 0.05). Correlation analyses were carried out using Pearson’s correlation test to examine interrelation between levels of plasma sex steroids and expression of *lep* and *lepr* in spleen and head kidney during reproductively active and quiescent phases in male as well as female *C. punctata*. Also, a correlation between *lep* and *lepr* expression was analysed (Spearman’s correlation test at 95% confidence interval) in each lymphoid tissue of male as well as female along their reproductive phases. The statistical analyses were carried out using GraphPad Prism5 software.

## Supplementary information

Supplementary information.

## Data Availability

The data of the present study would be made available on reasonable request.
