# Supplementary material for: Reproductive phase-dependent variation, sexually dimorphic expression and sex steroids-mediated transcriptional regulation of lep and lepr in lymphoid organs of Channa punctata
Source: Sci Rep. 2020 Jan 22;10:999. doi: 10.1038/s41598-020-57922-x (PMC6976713; doi:10.1038/s41598-020-57922-x)
Supplement: Supplementary file 1 — Supplementary information. [file 41598_2020_57922_MOESM1_ESM.pdf]

Reproductive phase-dependent variation, sexually dimorphic expression and sex steroids-mediated transcriptional regulation of *lep* and *lepr* in lymphoid organs of *Channa punctata*

Amrita Bakshi & Umesh Rai*
Address: Department of Zoology, University of Delhi, Delhi 110007, INDIA
Correspondence and requests for materials should be addressed to *U.R. (e-mail: rai_u@rediffmail.com)

Supplementary Table S1. Primer sequences (5'→3') used for reverse transcriptase PCR (RT-PCR) of *lep* (leptin) and *lepr* (leptin receptor) in *C. punctata*

Primer	Primer sequence	Amplicon length (bp)	Annealing temperature (°C)
lep (forward)	CTTCACTTACTGACTGCGGG	309	59.4
lep (reverse)	CTTTGGCCTCTGTTCAGTGC		
lepr (forward)	CCTACTGCGTGGATGGATT	674	59.9
lepr (reverse)	CAGGTGGTCAAAGTTGTCAG		

Supplementary Table S2. Primer sequences (5'→3') used for quantitative real-time PCR (qPCR) of *lep* and *lepr* in *C. punctata*

Primer	Primer sequence	Amplicon length (bp)	Annealing temperature (°C)	% efficiency	Slope	r^2
lep (forward)	GGTGGTCAGGCTCAACAAAG	150	59.2	110.1	-3.101	0.99
lep (reverse)	ACCTGGGAGACACCGTTAAA					
lepr (forward)	CAGTGGTTACCTCAGACGCA	91	55.8	105.9	-3.189	0.99
lepr (reverse)	CAGGCCGGTCAGTGTAAGAA					
18S (forward)	CTGAACTGGGGCCATGATT	100	57.4	100	-3.32	1
18S (reverse)	CTTTCGCTTTCGTCCGTCT					

Supplementary Table S3. Spearman's rank correlation coefficient (ρ) ($p < 0.05$) between the expression of ligand (*lep*) and its receptor (*lepr*) in spleen and head kidney during different reproductive phases of male and female *C. punctata*

Tissue	Sex	Reproductive phase			
		Resting	Preparatory	Spawning	Post spawning
Spleen	Male	0	0.04762	-0.2143	-0.07143
	Female	0.01205	-0.04762	-0.6228	-0.4048
Head kidney	Male	0	-0.04762	-0.2619	0.3571
	Female	-0.1905	-0.6905	0.3810	0.02381
